# Supplementary figures and images for: An evolutionary conserved detoxification system for membrane lipid–derived peroxyl radicals in Gram-negative bacteria
Source: PLoS Biol. 2022 May 17;20(5):e3001610. doi: 10.1371/journal.pbio.3001610 (PMC9113575; doi:10.1371/journal.pbio.3001610)

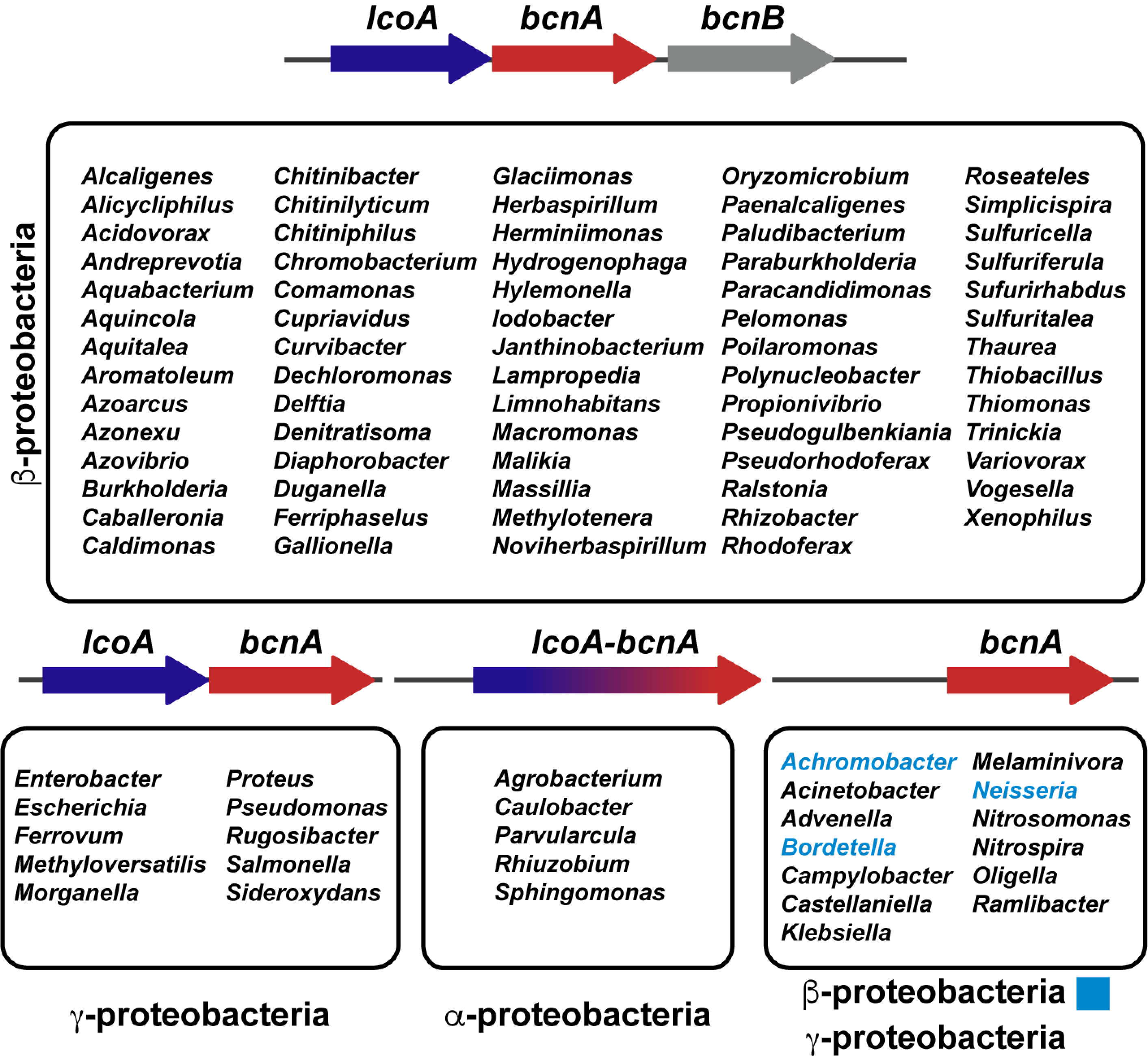

Supplement: S1 Fig — The analysis was performed by MultiGeneBlast as described in Materials and methods. Genes are not drawn to scale. The β-proteobacteria members in which bcnA is monocistronic are indicated in cyan. (TIF) [file pbio.3001610.s001.tif]

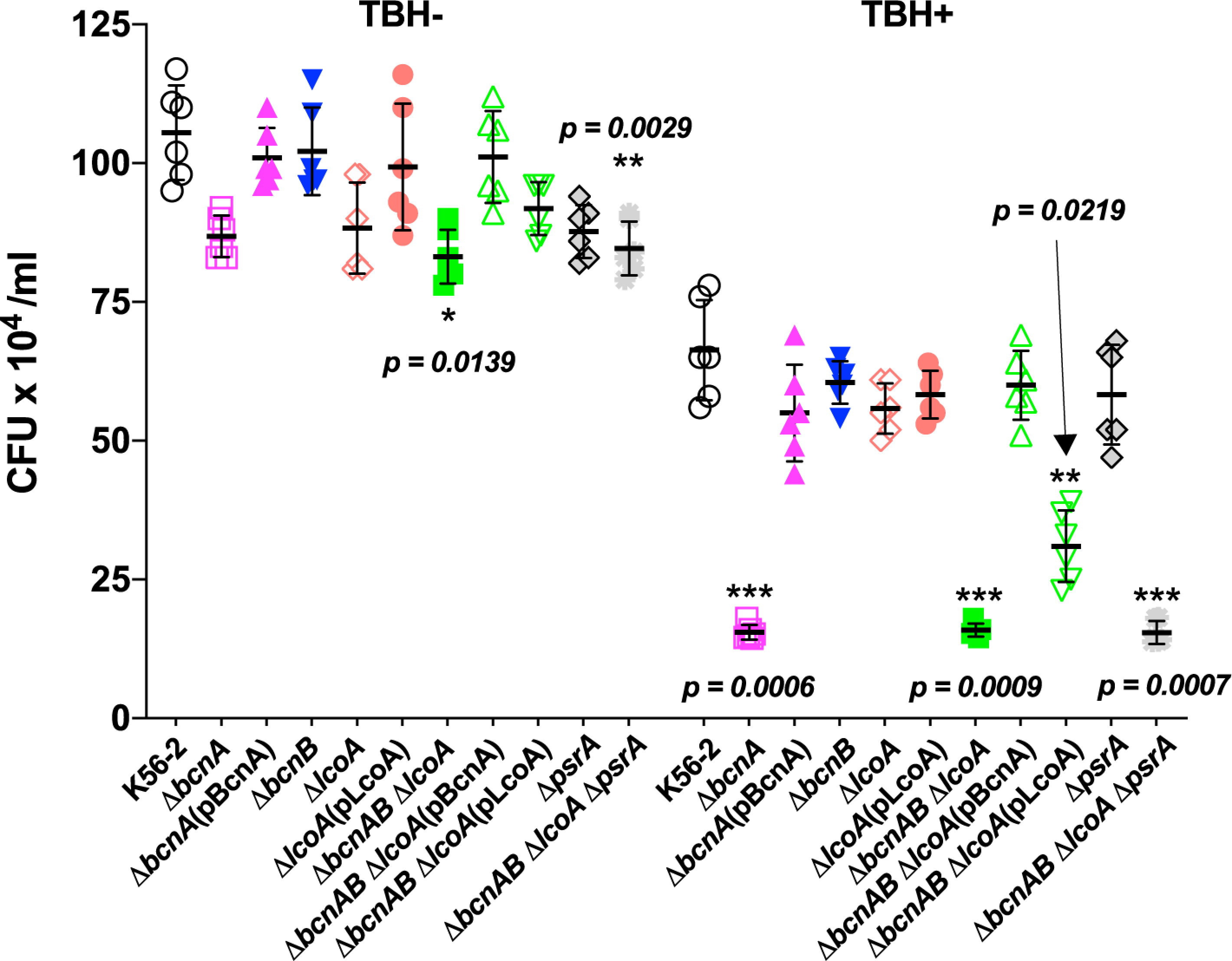

Supplement: S2 Fig — Bacterial cultures were challenged with either 30 μM (final concentration) tert-butyl-hydroperoxide (TBH+) or vehicle control (TBH−) for 1 hour at 37°C. Results are shown as the mean CFU/ml ± SD of surviving bacteria from 3 independent biological replicates in duplicate. p-Values relative to the CFU/ml recovered from the wild-type strain K56-2 were calculated by 2-way ANOVA with the Sidak multiple comparison test. *, p ≤ 0.05; **, p≤0.01 ***, p ≤0.0001. When significant, the calculated p-values are shown. Data underlying the graph in this figure can be found in S1 Data. CFU, colony-forming unit; SD, standard deviation; TBH, tert-butyl hydroperoxide. (TIF) [file pbio.3001610.s002.tif]

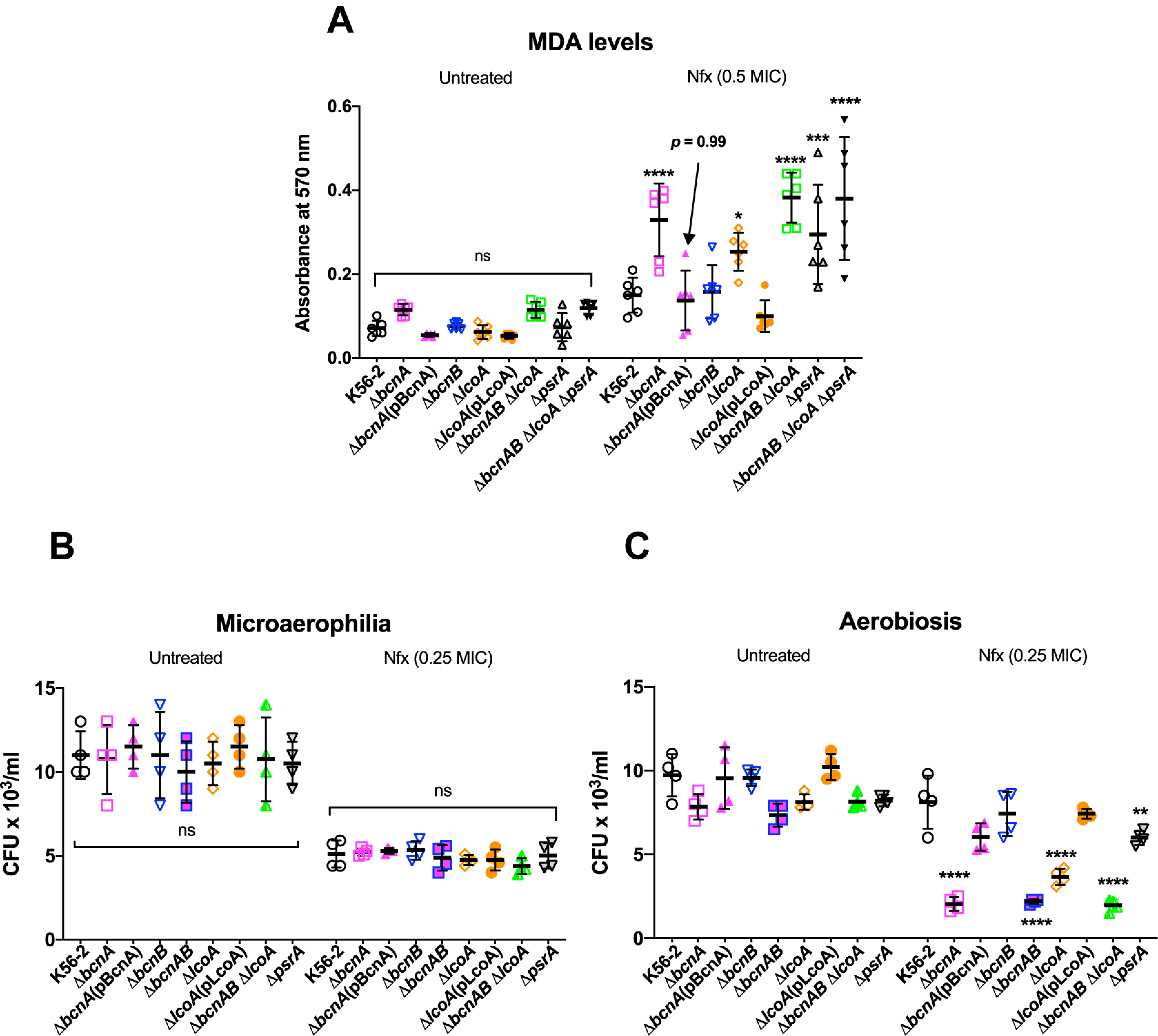

Supplement: S3 Fig — K56-2 and mutants carried the control plasmid vector pDA17 or the indicated complementing plasmid (in parentheses). (A) Bacteria were grown overnight with or without Nfx at concentrations corresponding to the 0.5 MIC (32 μg ml−1 for the wild-type strain K56-2, ΔbcnB, ΔlcoA(pLcoA), and ΔpsrA, 24 μg ml−1 for ΔbcnA(pBcnA), 16 μg ml−1 for ΔlcoA, 4 μg ml−1 for ΔbcnA, and ΔbcnABΔlcoA). Results are shown as the mean of absorbance at 570 nm ± SD. Data represent the results of 3 independent experiments, each done in duplicate. The statistical significance of MDA levels untreated bacteria was determined by 2-way ANOVA. Strains comparisons were made against the wild-type K56-2 by Dunnett post hoc analysis. (B) Bacterial cultures were diluted and plated on LB plates with or without Nfx (at 0.25 MIC) and incubated in anaerobic jars under microaerophilic conditions. CFU were enumerated after 24-hour incubation at 37°C. Results are shown as the mean CFU/ml ± SD from 2 independent biological replicates in duplicate. (C) Control experiment as in (B), but under standard aerobic conditions. Results are shown as the mean CFU/ml ± SD from 2 independent biological replicates in duplicate. The statistical significance of results in panels A–C was determined by 2-way ANOVA. Individual comparisons were made against K56-2 by Dunnett post hoc analysis. *, p ≤ 0.05, **; p ≤ 0.01; ***, p ≤ 0.001; ****, p ≤0.0001; ns, nonsignificant. Data underlying the graphs in this figure can be found in S1 Data. CFU, colony-forming unit; MDA, malondialdehyde; MIC, minimum inhibitory concentration; Nfx, norfloxacin; SD, standard deviation. (TIF) [file pbio.3001610.s003.tif]

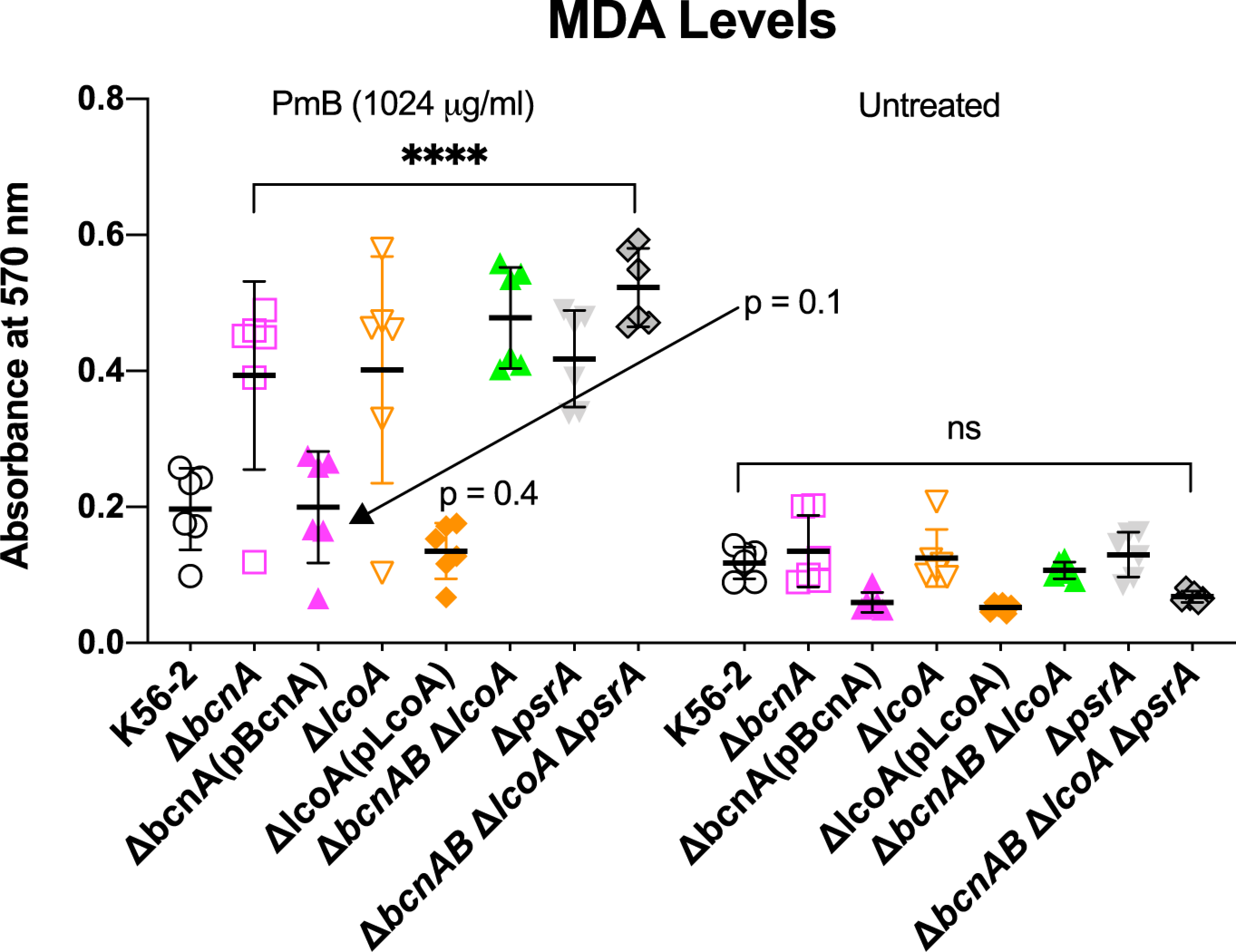

Supplement: S4 Fig — Wild-type strains and mutants contained the control plasmid vector pDA17 or the indicated complementing plasmid (in parentheses). Bacteria were grown overnight with or without 1,024 μg ml−1 PmB. MDA was determined by ELISA (see Materials and methods). Results are shown as the mean of absorbance at 570 nm ± SD. Data represent the results of 3 independent experiments, each done in duplicate. The statistical significance of MDA levels obtained from PmB-treated versus untreated bacteria was determined by 2-way ANOVA. Strains comparisons were made against the wild-type K56-2 by Dunnett multiple comparison test. ****, p ≤ 0.0001; ns, nonsignificant. Data underlying the graph in this figure can be found in S1 Data. MDA, malondialdehyde; PmB, polymyxin B; SD, standard deviation. (TIF) [file pbio.3001610.s004.tif]

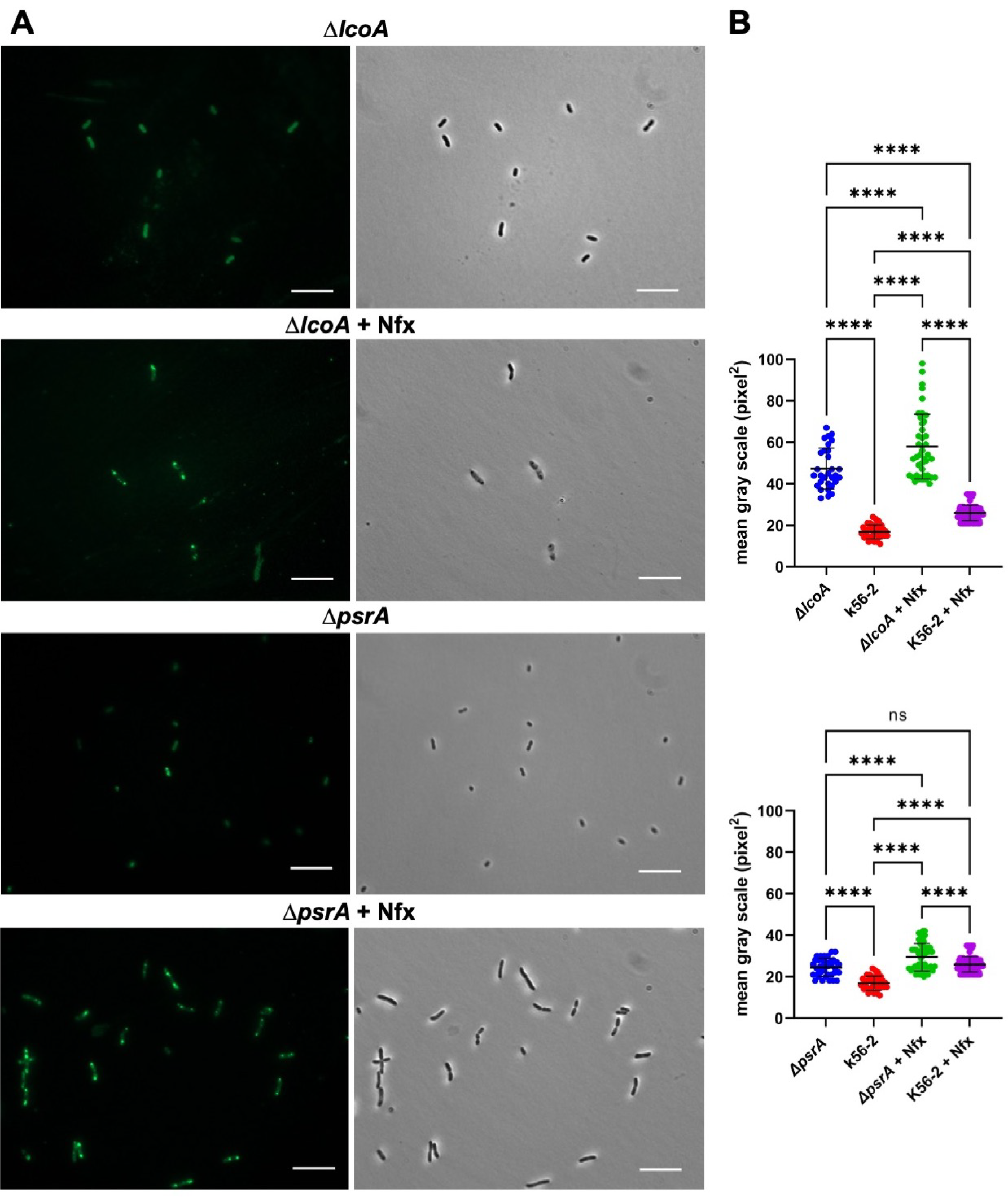

Supplement: S5 Fig — (A) Fluorescence (left) and phase-contrast (right) images of ΔlcoA and ΔpsrA strains untreated or treated with 75% MIC for Nfx and incubated with NBD-Pen. Scale bars, 10 μm. (B) Quantitative analysis of fluorescence intensity based on fluorescence images in panel A. SD is shown as error bars, and mean values are shown as horizontal black lines. Statistical significance was determined by 1-way ANOVA with Tukey multiple comparisons test’s post hoc analysis. ****, p < 0.0001; **, p < 0.005; ns, nonsignificant. Data underlying the graph in this figure can be found in S2 Data. NBD-Pen, 2,2,6-trimethyl-4-(4-nitrobenzo [1,2,5]oxadiazol-7-ylamino)-6-pentylpiperidine-1-oxyl; Nfx, norfloxacin; SD, standard deviation. (TIF) [file pbio.3001610.s005.tif]

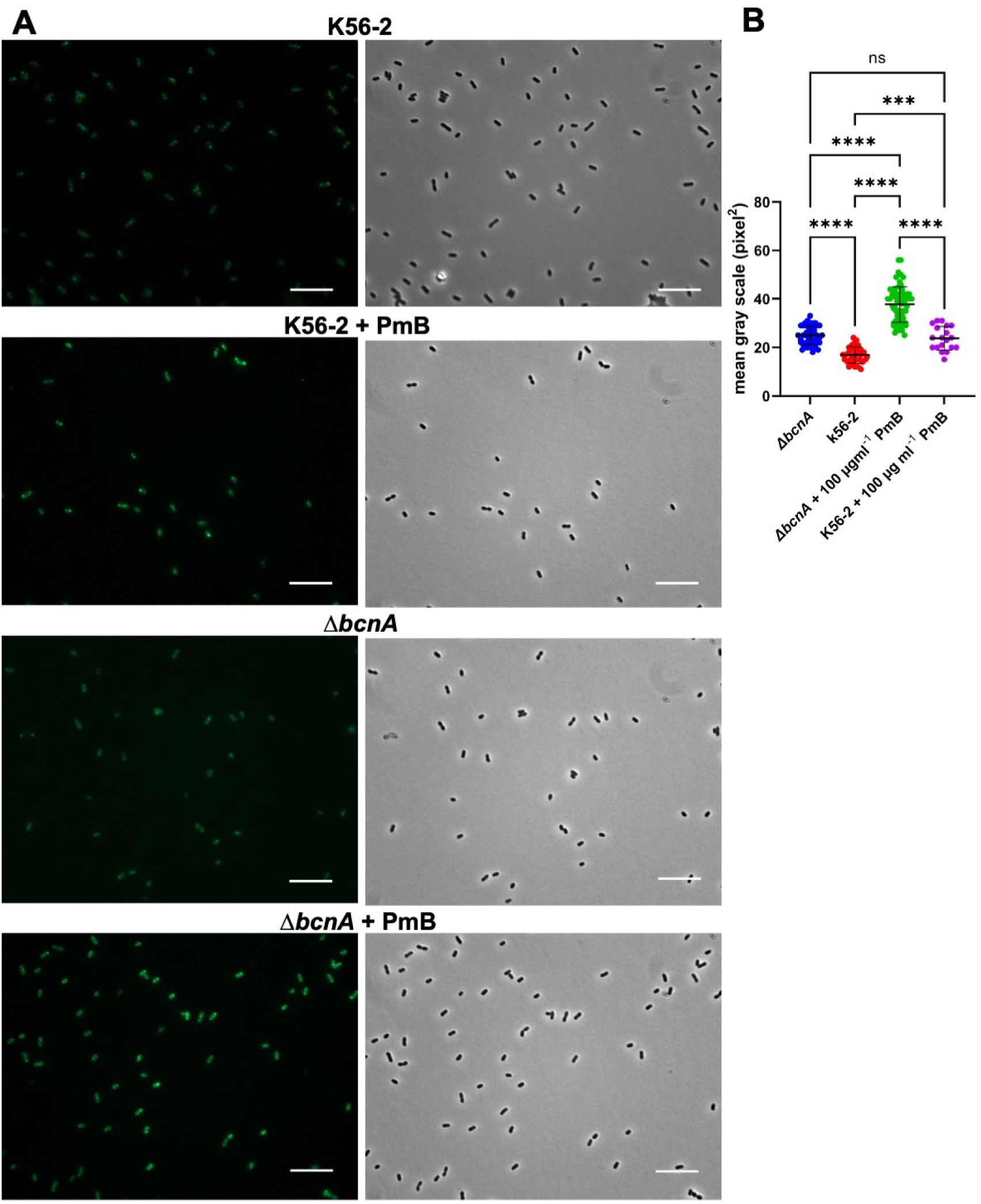

Supplement: S6 Fig — (A) Fluorescence (left) and phase-contrast (right) images of K56-2 and ΔbcnA strains untreated or treated with 100 μg ml−1 of PmB and incubated with NBD-Pen. Scale bars, 10 μm. (B) Quantitative analysis based on fluorescence images in panel A. SD is shown as error bars, and mean values are shown as horizontal black lines. Statistical significance was determined by 1-way ANOVA with Tukey multiple comparisons test’s post hoc analysis. ****, p < 0.0001; ***; p < 0.001; **, p < 0.005; ns, no significative. Data underlying the graph in this figure can be found in S2 Data. NBD-Pen, 2,2,6-trimethyl-4-(4-nitrobenzo [1,2,5]oxadiazol-7-ylamino)-6-pentylpiperidine-1-oxyl; PmB, polymyxin B; SD, standard deviation. (TIF) [file pbio.3001610.s006.tif]

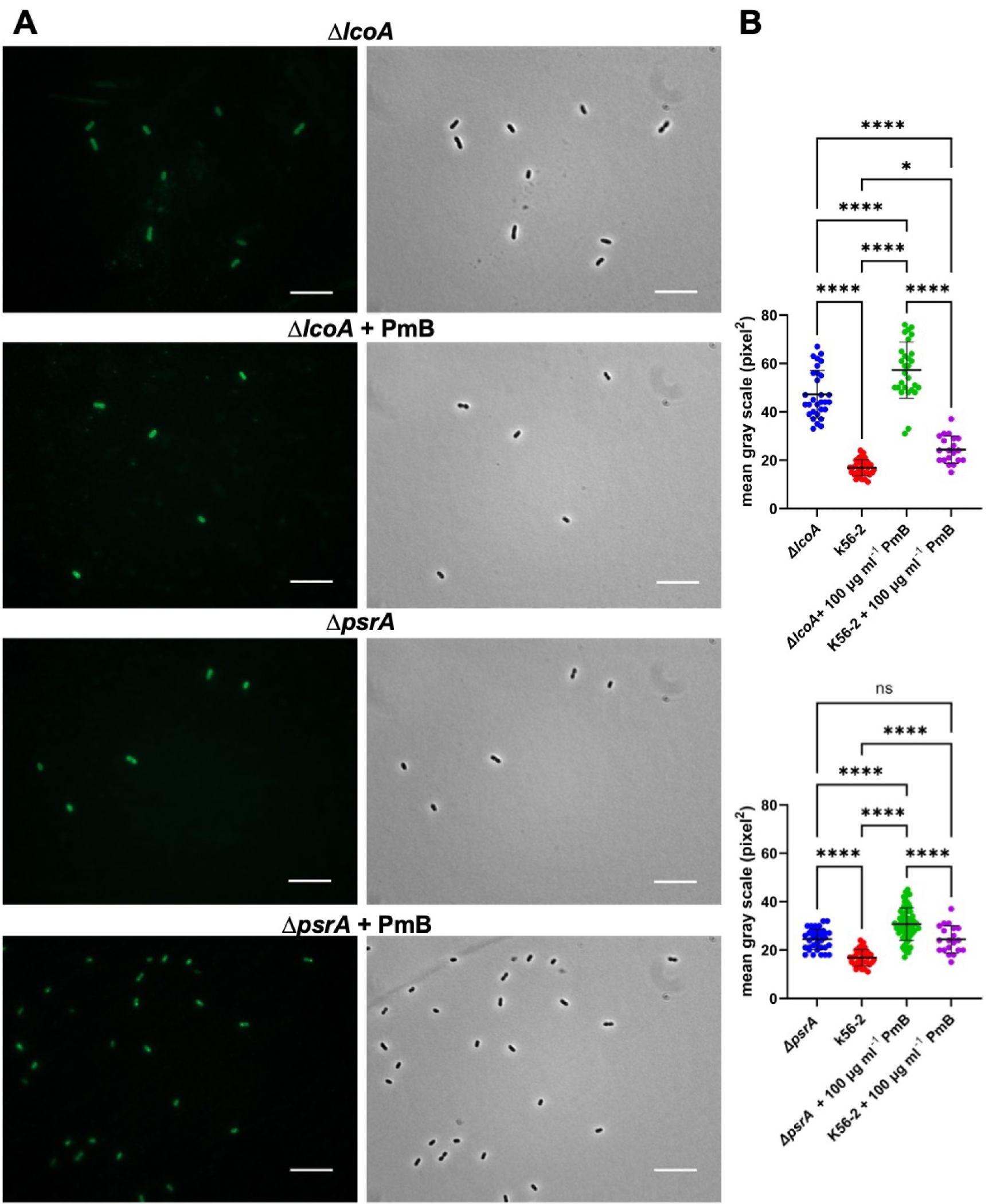

Supplement: S7 Fig — (A) Fluorescence (left) and phase-contrast (right) images of ΔlcoA and ΔpsrA strains untreated or treated with 100 μg ml−1 of PmB and incubated with NBD-Pen. Scale bars, 10 μm. (B) Quantitative analysis based on fluorescence images in panel A. SD is shown as error bars, and mean values are shown as horizontal black lines. ****, p < 0.0001, ns, no significative. Data underlying the graph in this figure can be found in S2 Data. NBD-Pen, 2,2,6-trimethyl-4-(4-nitrobenzo [1,2,5]oxadiazol-7-ylamino)-6-pentylpiperidine-1-oxyl; PmB, polymyxin B. (TIF) [file pbio.3001610.s007.tif]

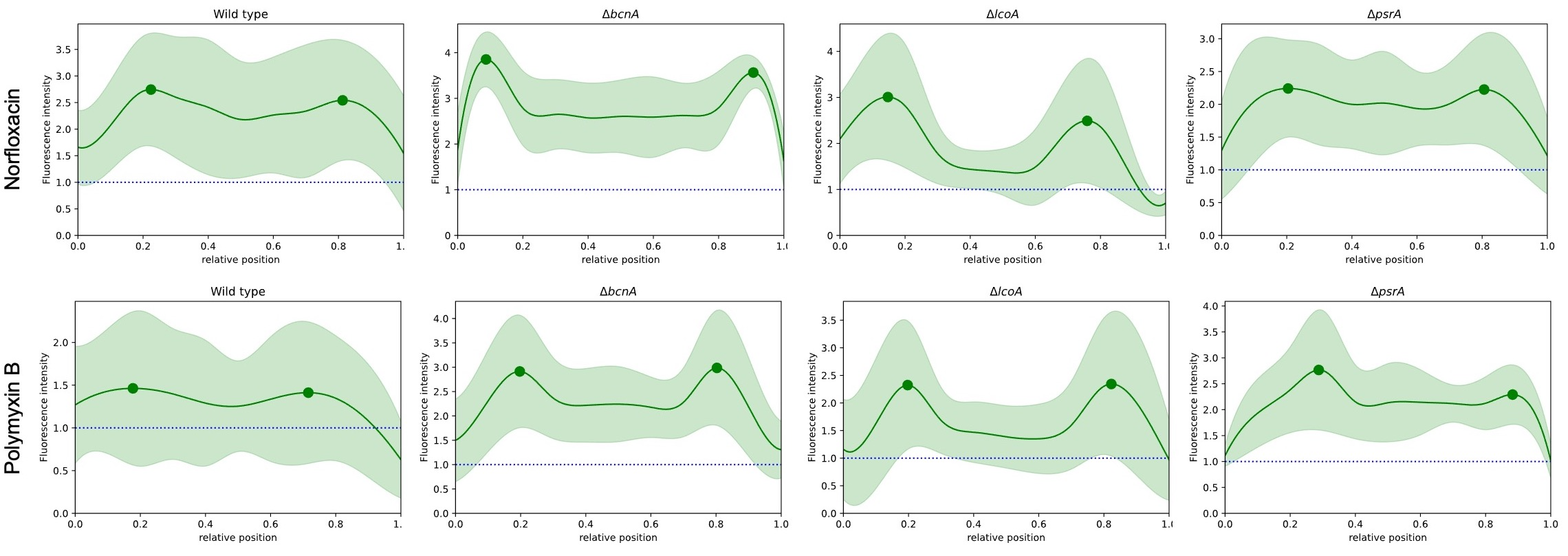

Supplement: S8 Fig — Averaged green intensity profiles of cells labeled with NBD-Pen versus normalized cell length. The intensities along the long axis of each cell were averaged. The average value is depicted as a green line for each strain. The shaded space surrounding the NBD-Pen fluorescence intensity profiles designates the SD of the intensity at each point. The peak of fluorescent intensity produced in the treated bacteria is shown as green dots. Data underlying the graph in this figure can be found in S2 Data. NBD-Pen, 2,2,6-trimethyl-4-(4-nitrobenzo [1,2,5]oxadiazol-7-ylamino)-6-pentylpiperidine-1-oxyl; SD, standard deviation. (TIF) [file pbio.3001610.s008.tif]

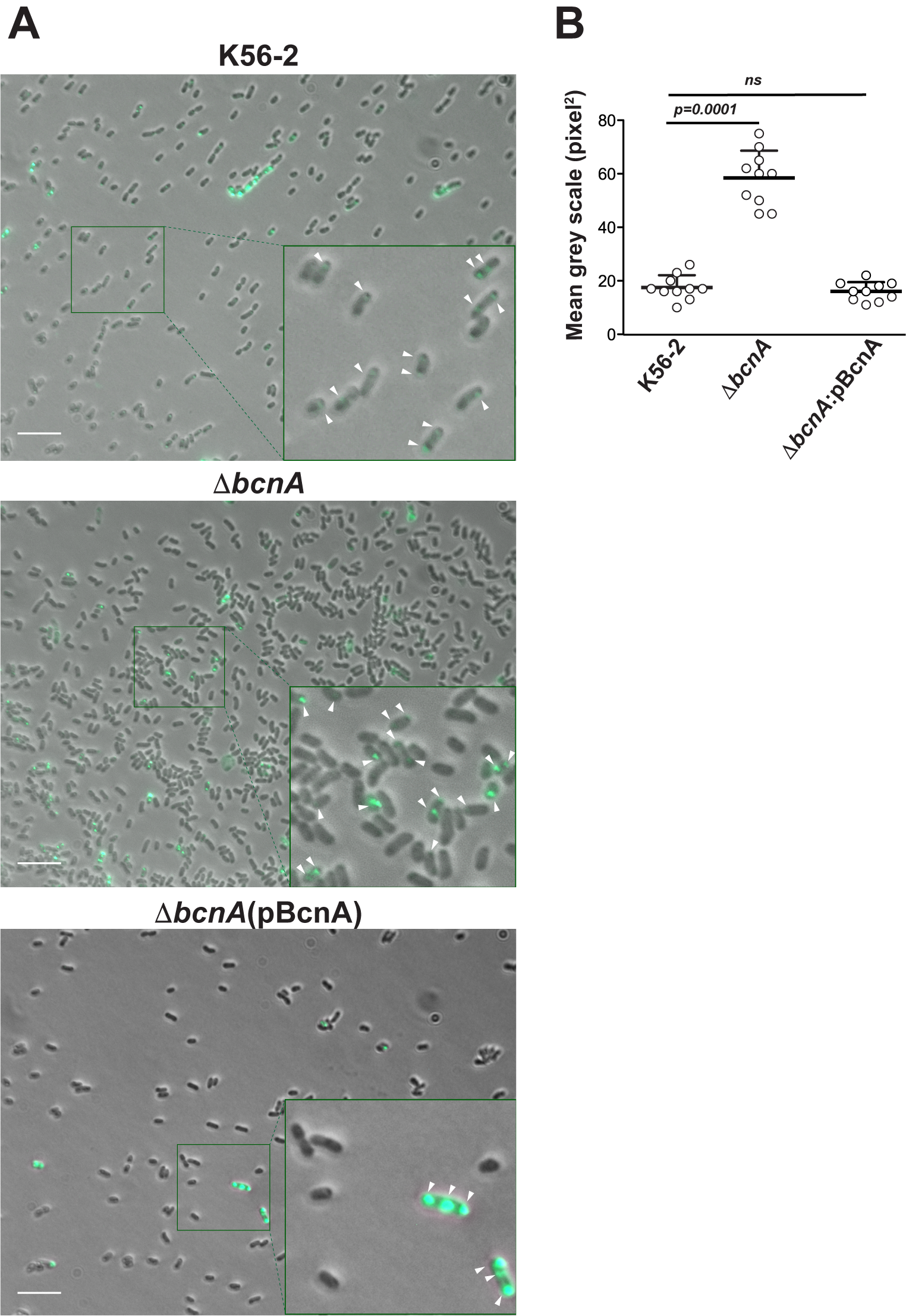

Supplement: S9 Fig — (A) Overlay fluorescence and phase contrast images of bacterial cells incubated with 1 μM H4BPMHC for 15 minutes at 37°C. The insets are zoomed sections of the images to provide more detail of the location of the fluorescent at the cell poles and mid cell (arrowheads). Scale bars, 5 μm. (B) Quantitative analysis of the fluorescent intensities of all bacterial cells; the results represent the mean ± SD from 3 independent experiments. Statistical significance was calculated by 1-way ANOVA with Tukey multiple comparisons test’s post hoc analysis. ns, nonsignificant. Data underlying the graph in this figure can be found in S1 Data. H4BPMHC, 8-((6-hydroxy-2,5,7,8-tetramethylchroman-2-yl)-methyl)-1,5-di(3-chloropropyl)-pyrromethene fluoroborate; SD, standard deviation. (TIF) [file pbio.3001610.s009.tif]

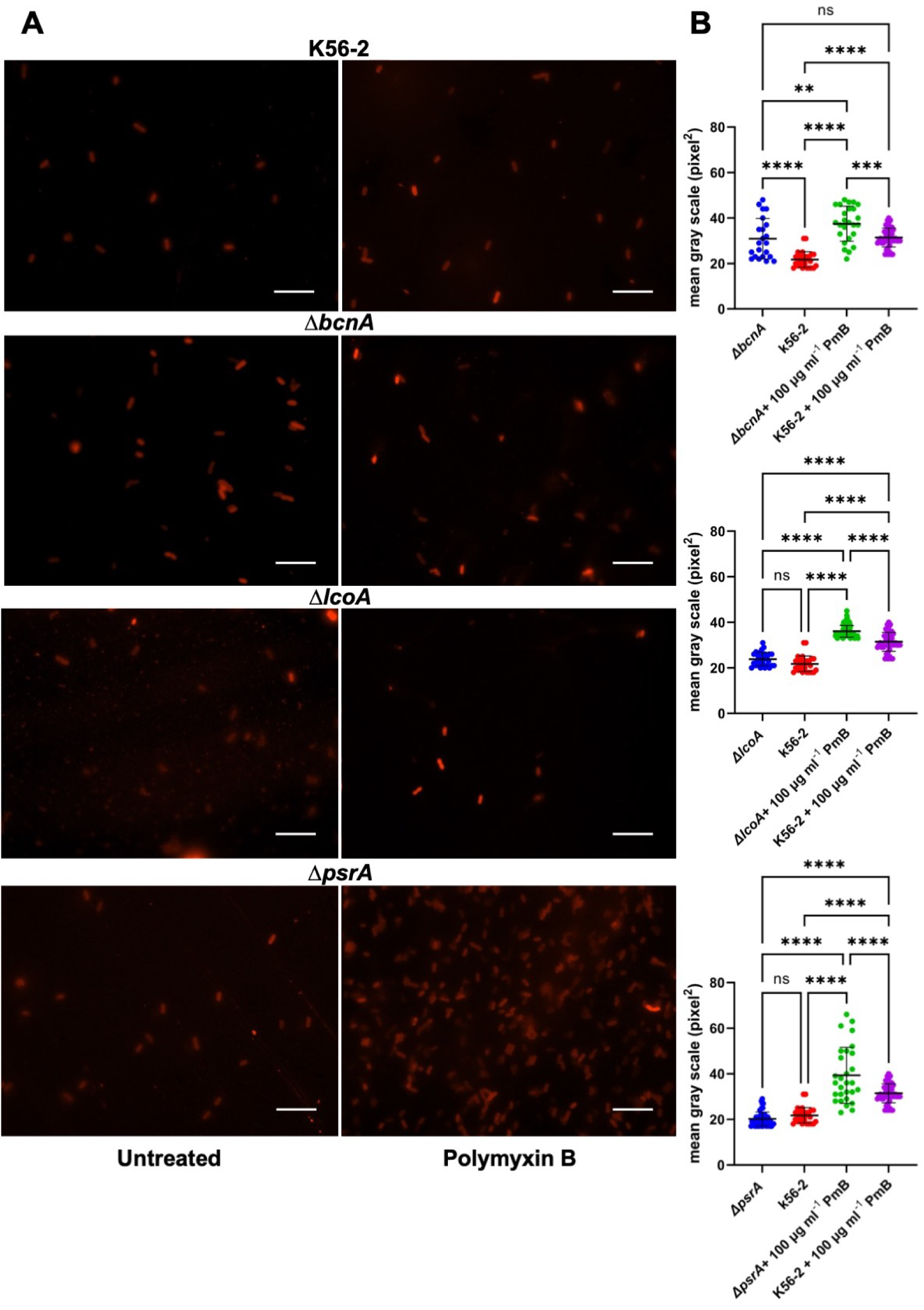

Supplement: S10 Fig — (A) Detection of CL using 1 μM NAO for 15 minutes at room temperature. Bacteria were examined with a Zeiss microscope using a 100× oil immersion lens with an excitation at 640 nm. Scale bars, 10 μm. (B) Quantification of fluorescence intensity by ImageJ; the results represent the mean ± SD from 3 independent experiments. The statistical significance of the relative mean grayscale levels obtained from treated (PmB) versus untreated bacteria was determined by 1-way ANOVA with Tukey multiple comparisons test’s post hoc analysis. ****, p < 0.0001; ***; p < 0.001; **, p < 0.005; ns, nonsignificant. Data underlying the graph in this figure can be found in S2 Data. CL, cardiolipin; NAO, Acridine Orange 10-nonyl bromide; PmB, polymyxin B; SD, standard deviation. (TIF) [file pbio.3001610.s010.tif]

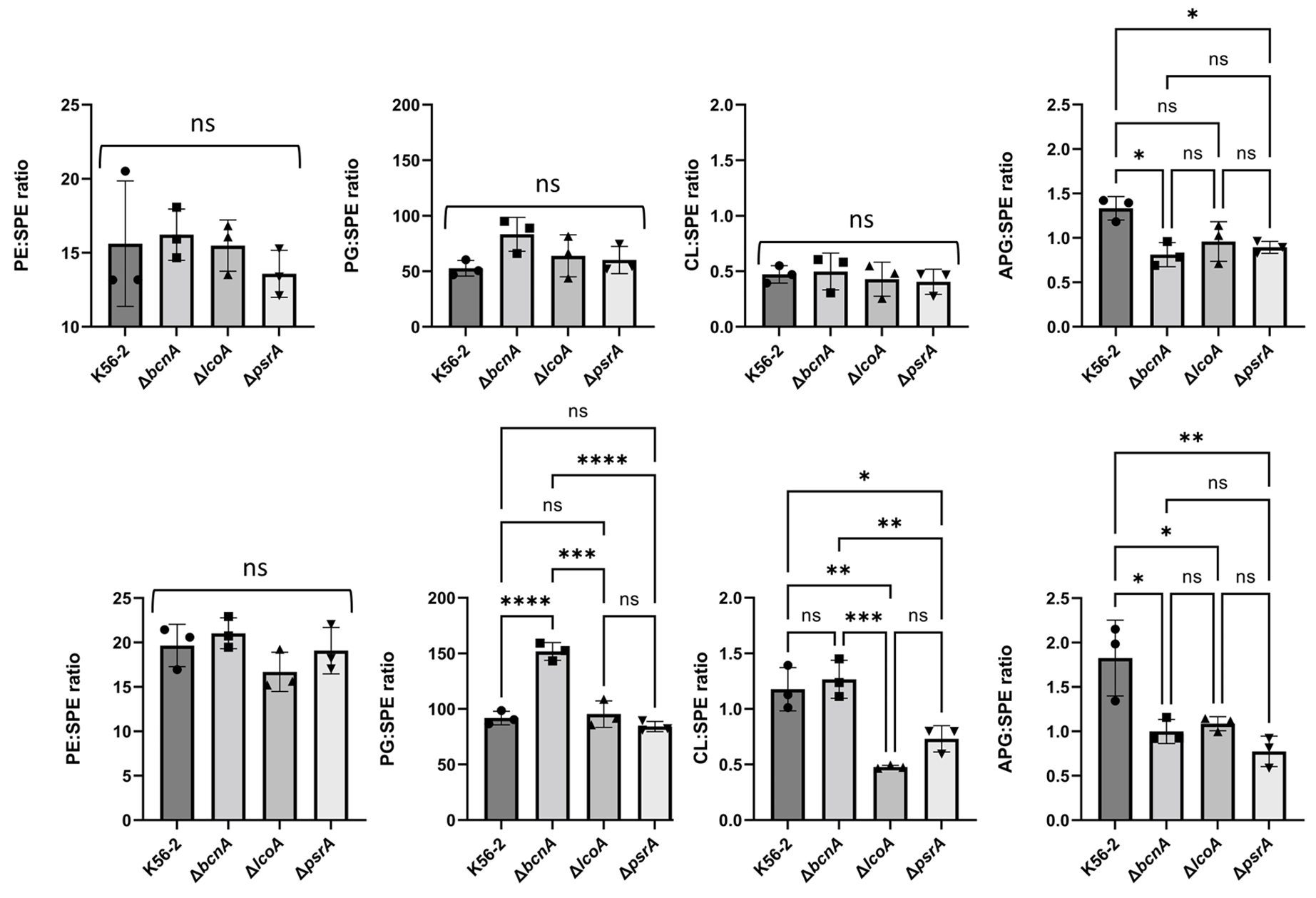

Supplement: S11 Fig — CL, PG, PE, and 2′ APG were identified and quantified by mass spectrometry using QuantAnalysis (Bruker Daltonics), which calculated the area under each peak for each sample. The sum of these areas was compared to that of the known lipid standard, SPE. (A) Lipids from bacteria under no antibiotic treatment. (B) Lipids from bacteria treated with 1,024 μg ml−1 of PmB. SD is shown as error bars, and mean values are shown as bars. ****, p < 0.0001; ***; p < 0.001; **, p < 0.005; *, p < 0.05; ns, not significant. Data underlying the graphs in this figure can be found in S2 Data. APG, alanyl-phosphatidylglycerol; CL, cardiolipin; PE, phosphatidylethanolamine; PG, phosphatidylglycerol; PmB, polymyxin B; SD, standard deviation; SPE, sphingosyl phosphatidylethanolamine. (TIF) [file pbio.3001610.s011.tif]
