# Supplementary material for: An evolutionary conserved detoxification system for membrane lipid–derived peroxyl radicals in Gram-negative bacteria
Source: PLoS Biol. 2022 May 17;20(5):e3001610. doi: 10.1371/journal.pbio.3001610 (PMC9113575; doi:10.1371/journal.pbio.3001610)
Supplement: S1 Table — (DOCX) [file pbio.3001610.s012.docx]

**S1 Table. Reagents.**

| **Reagent** | **Source** | **Catalog #/Reference** |
| --- | --- | --- |
| Acetic acid, Glacial, Pharma grade | Sigma Aldrich | ARK2183 |
| Acetone, HPLC grade | Sigma Aldrich | 34850 |
| Acridine Orange 10-nonyl bromide (NAO) | Invitrogen | A1372 |
| Bovine Serum Albumin, Bioreagent grade | Sigma Aldrich | A9418 |
| Dimethyl sulfoxide (DMSO), ACSC reagent | Sigma Aldrich | 472301 |
| 3′,6-dinonyl neamine tetratrifluoroacetate (diNn) | Synthesized by Jean-Luc Décout | [1] |
| Diphenyl-1-pyrenylphosphine (DPPP) | Invitrogen | D7894 |
| Gentamicin sulfate | Sigma Aldrich | G3632 |
| H_4_BPMHC; 8-((6-hydroxy-2,5,7,8-tetramethylchroman-2-yl)-methyl)-1,5-di(3-chloropropyl)-pyrromethene fluoroborate | Synthesized by Julia McCain | [2] |
| Hydrochloric acid (HCl) | Sigma-Aldrich | 320331 |
| Hexadecane, Reagent Plus | Sigma-Aldrich | H6703 |
| Polymyxin B, sulfate salt | Sigma Aldrich | P4932 |
| Potassium Tellurite | Sigma Aldrich | P0677 |
| Phosphate-buffered solution, pH 7.4 | Gibco | 10010023 |
| Protease Inhibitor, cOmplete ULTRA Tablets, Mini, *EASYpack* Protease Inhibitor Cocktail | Merck, Sigma Aldrich | 5892970001 |
| *tert*-butyl hydroperoxide (TBH), 70 wt. % in H_2_O | Sigma Aldrich | 458139 |
| Tetracycline hydrochloride | Sigma Aldrich | T3383 |
| 3,3',5,5'-tetramethylbenzidine (TMB) | Sigma-Aldrich | 860336 |
| Trimethoprim, HPLC grade | Sigma Aldrich | T7883 |
| 2,2,6-trimethyl-4-(4-nitrobenzo [1,2,5]oxadiazol-7-ylamino)-6-pentylpiperidine-1-oxyl (NBD-Pen) | Aobious | AOB9999 |
| Tween20 | Sigma Aldrich | P1379 |

1. Zimmermann L, Bussière A, Ouberai M, Baussanne I, Jolivalt C, Mingeot-Leclercq MP, et al. Tuning the antibacterial activity of amphiphilic neamine derivatives and comparison to paromamine homologues. J Med Chem. 2013;56(19):7691-705. Epub 2013/10/03. doi: 10.1021/jm401148j. PubMed PMID: 24083676.

2. Greene LE, Lincoln R, Cosa G. Rate of lipid peroxyl radical production during cellular homeostasis unraveled via fluorescence imaging. J Am Chem Soc. 2017;139(44):15801-11. Epub 2017/10/19. doi: 10.1021/jacs.7b08036. PubMed PMID: 29043787.
