# Supplementary material for: An evolutionary conserved detoxification system for membrane lipid–derived peroxyl radicals in Gram-negative bacteria
Source: PLoS Biol. 2022 May 17;20(5):e3001610. doi: 10.1371/journal.pbio.3001610 (PMC9113575; doi:10.1371/journal.pbio.3001610)
Supplement: S2 Table — (DOCX) [file pbio.3001610.s013.docx]

**S2 Table. Bacterial strains and plasmids.**

**Strains Relevant Characteristics^a^** **Source**

**or plasmid or reference**

**Strains**

***Burkholderia cenocepacia***

K56-2 ET12 clone related to J2315, CF clinical isolate [1]

Δ*bcnA* K56-2; ΔK562_13237 (ΔBCAL3311)^b^ [2]

Δ*bcnB*  K56-2; ΔK562_13236 (ΔBCAL3310)^b^ [2]

Δ*bcnAB* K56-2; ΔK562_13237 ΔK562_13236 [3]

Δ*lcoA* K56-2; ΔK562_13238 (ΔBCAL3312)^b^ [4]

Δ*bcnABΔlcoA* Δ*bcnAB*; ΔK562_13238 [4]

Δ*psrA* K56-2; ΔK562_20604 (ΔBCAM0596)^b^ This study

Δ*bcnAB*Δ*lcoA*Δ*psrA* Δ*bcnABΔlcoA;* ΔK562_20604 This study

***Escherichia coli***

DH5α *F^-^* Φ*80lacZ M15 endA1 recA1 supE44 hsdR17(r_K_^-^ m_K_^+^)*

*deoR thi-1 nupG supE44 gyrA96relA1* Δ*(lacZYA-argF)*

*U169, λ^-^* Lab stock

GT115 *F^-^ mcrA*Δ*(mrr-hsdRMS-mcrBC)* Φ*80*Δ*lac*ZΔM15 Δ*lacX74*

*recA1 rpsL(StrA) endA1* Δ*dcm uidA(*Δ*MluI)::pir116*

Δ*sbcC-sbcD* Invivogen

**Plasmids**

pDA17 ori_pBBRI_, Tet^R^, mob+, P_dhfr_, FLAG epitope D. Aubert

pDAI-SceI-SacB ori_pBBR1_, Tet^R^, Pdhfr, mob+, expressing I-SceI, SacB [5]

pGPI-SceI ori_R6K_, Tp^R^, mob^+^, including an I-SceI restriction site [6]

pRK2013 ori_ColE1_, RK2 derivative, Kan^R^, mob^+^, tra^+^ [7]

pExp*bcnA* *bcnA* gene without signal peptide cloned in pET28a(+) [3]

pBcnA pDA17 harbouring *bcnA* gene with C-terminus FLAG, Tet^R^ [2]

pLcoA pDA17 harbouring *lcoA* gene, Tet^R^ This study

pDel*psrA* pGPI-SceI with fragments flanking K562_20604, Tp^R^ This study

pYqhD pDA17 harbouring *yqhD* gene, Tet^R^ This study

pPsrA pDA17 harbouring *psrA* gene, Tet^R^ This study

^a^ The K56-2 loci identification can be found in [GenBank accession CP053301](https://www.ncbi.nlm.nih.gov/nuccore/CP053301) [8]. The BCAL/BCAM loci indicate homologous genes in the related strain J2315, which was used as a reference *B. cenocepacia* ET-12 before the completion of the K56-2 genome [8]. Tet^R^, Tetracycline resistance, Tp^R^, trimethoprim resistance, Kan^R^, kanamycin resistance.

^b^ Denotes the equivalent annotation for the *B. cenocepacia* type strain J2315 [9].

**References**

1. Mahenthiralingam E, Coenye T, Chung JW, Speert DP, Govan JR, Taylor P, et al. Diagnostically and experimentally useful panel of strains from the *Burkholderia cepacia* complex. J Clin Microbiol. 2000;38(2):910-3. PubMed PMID: 10655415.

2. El-Halfawy OM, Klett J, Ingram RJ, Loutet SA, Murphy MEP, Martín-Santamaría S, et al. Antibiotic capture by bacteriocalins uncovers an extracellular mechanism of intrinsic antibiotic resistance. mBio. 2017;Mar 14;8(2).

3. El-Halfawy OM, Valvano MA. Chemical communication of antibiotic resistance by a highly resistant subpopulation of bacterial cells. PLoS One. 2013;8(7):e68874. doi: 10.1371/journal.pone.0068874. PubMed PMID: 23844246; PubMed Central PMCID: PMC3700957.

4. Naguib MM, Valvano MA. Vitamin E increases antimicrobial sensitivity by inhibiting bacterial lipocalin antibiotic binding. mSphere. 2018;3(6). Epub 2018/12/14. doi: 10.1128/mSphere.00564-18. PubMed PMID: 30541778; PubMed Central PMCID: PMCPMC6291622.

5. Hamad MA, Skeldon AM, Valvano MA. Construction of aminoglycoside-sensitive *Burkholderia cenocepacia* strains for use in studies of intracellular bacteria with the gentamicin protection assay. Appl Environ Microbiol. 2010;76(10):3170-6. Epub 2010/03/30. doi: AEM.03024-09 [pii]

10.1128/AEM.03024-09. PubMed PMID: 20348312; PubMed Central PMCID: PMC2869153.

6. Flannagan RS, Linn T, Valvano MA. A system for the construction of targeted unmarked gene deletions in the genus *Burkholderia*. Environ Microbiol. 2008;10:1652-60.

7. Figurski DH, Helinski DR. Replication of an origin-containing derivative of plasmid RK2 dependent on a plasmid function provided in *trans*. Proc Natl Acad Sci USA. 1979;76(4):1648-52.

8. Garcia-Romero I, Valvano MA. Complete genome sequence of *Burkholderia cenocepacia* K56-2, an opportunistic pathogen. Microbiol Resour Announc. 2020;9(43). Epub 2020/10/24. doi: 10.1128/MRA.01015-20. PubMed PMID: 33093048; PubMed Central PMCID: PMCPMC7585848.

9. Holden M, Seth-Smith H, Crossman L, Sebaihia M, Bentley S, Cerdeno-Tarraga A, et al. The genome of *Burkholderia cenocepacia* J2315, an epidemic pathogen of cystic fibrosis patients. J Bacteriol. 2009;191:261-77.
