# Supplementary material for: An evolutionary conserved detoxification system for membrane lipid–derived peroxyl radicals in Gram-negative bacteria
Source: PLoS Biol. 2022 May 17;20(5):e3001610. doi: 10.1371/journal.pbio.3001610 (PMC9113575; doi:10.1371/journal.pbio.3001610)
Supplement: S3 Table — (DOCX) [file pbio.3001610.s014.docx]

**S3 Table. Primers.**

| Primers | Oligonucleotide sequence, 5'–3'^a^ | Restriction enzyme |
| --- | --- | --- |
| Construction of the mutagenic plasmid for the Δ*psrA* deletion mutant (ΔK562_20604) | Forward 1a: TTTTGAATTCCCCATATCCACGTGTGTTGC  Reverse 1b: TTTTCTCGAGCTGCGCTTCGACACGGTC  Forward 2a: TTTTCTCGAGCGCAACTTCATCGGCACCC  Reverse 2b: TTTTTCTAGAGCGGAAGAACAGGAACGATT | *Eco*RI  *Xho*I  *Xho*I  *Xba*I |
| Cloning of the *lcoA* gene for complementation experiments | Forward: TTTTCATATGATGGCATCGAAATCGCCG  Reverse: TTTTTCTAGATCATTTGGCAAAGGGAAG | *Nde*I  *Xba*I |
| Cloning of the *psrA* gene for complementation experiments | TTTTCATATGATGAGCGACTGGCACTTCCACA  TTTTTCTAGAAGTGAAGCGCCCGCTCGGGC | *Nde*I  *Xba*I |
| Cloning of the *yqhD* gene for complementation experiments | TTTCATATGATGAACAACTTTAATCTGCACACCCCA  TTTTTCTAGAAGCGGGCGGCTTCGTATATAC | *Nde*I  *Xba*I |
| Flanking sequences to the multiple cloning sites of the pDA17 cloning vector for DNA sequencing | Forward: GGATTCGGAATTCTACATATGATGGCA  Reverse: GTCGTCCTTGTAGTCTCTAGATCATTT |  |

^a^ Sequences in colour denote the restriction endonuclease cut site and extra 5' nucleotides to facilitate restriction digestion.
