# Supplementary material for: An evolutionary conserved detoxification system for membrane lipid–derived peroxyl radicals in Gram-negative bacteria
Source: PLoS Biol. 2022 May 17;20(5):e3001610. doi: 10.1371/journal.pbio.3001610 (PMC9113575; doi:10.1371/journal.pbio.3001610)
Supplement: Attachment — Submitted filename: Response to Reviewers.pdf [file pbio.3001610.s017.pdf]

1 March 2022

Dario Ummarino, PhD
Senior Editor, PLOS Biology
dummarino@plos.org

Dear Dr. Ummarino

RE: PBIOLOGY-D-21-02956R1 – "An evolutionary conserved detoxification system for membrane lipid-derived peroxyl radicals in Gram-negative bacteria"

Thank you so much for your email on the 26th of January 2022 requesting us to provide a revised manuscript **(herein RV)** incorporating the comments from the reviewers and the editorial instructions.

Please find below our detailed response, point by point, to the reviewers' comments

Reviewer #1

We thank the Reviewer for acknowledging our manuscript *"is conceptually sound, and its findings may have applications to better understanding bacterial cell death and combatting antibiotic resistance"*. This reviewer also stated *"…this Report may certainly be of interest to PLOS Biology's microbiology audience, as well as other researchers who are interested in the role of reactive species in biology"*.

1. *Dose-response:*
We did not perform any of the suggested experiments following the request from the Editor. However, we would like to clarify to the reviewer that the experiments were planned with stressors at sublethal doses close to MIC, which vary according to the antibiotic/chemical used. This was done to create a similar level of stress as when bacterial death is approaching and where peroxidation and the proteins we are investigating play a clear role. We agree that dose responses would have been interesting perhaps to define at which point our proteins become critical to protect bacteria from peroxidation, but this will be addressed in future studies.

2. *Relationship to lethality: In Fig. 2A, DPPP=O intensities for potassium tellurite treatment are presented, but there is no information shown about corresponding CFU/mL values under this treatment condition, as was done with antibiotics (Fig. 4). Similarly for TBH treatment in Fig. S1. The authors should, wherever applicable, measure the CFU/mL values and compare them to the fluorescence (and potentially other) readouts to better substantiate the claim that lipid peroxidation contributes to lethality.*

The DPPP assay is done over 1-hour incubation. The generation time of *B. cenocepacia* under optimal growth conditions (LB medium) is 1 h. Bacterial survival is not an issue in this assay, which is designed to quantify membrane peroxidation. Concerning TBH **(see RV, S2 Fig)**, we measured CFU/ml.

3. *Positive controls: The addition of positive ROS controls would facilitate comparison to known stressors. Exogenous addition of ROS such as H2O2 should lead to lipid peroxidation, which ought to be captured by the various probes the authors use. The authors might consider adding such a positive control to at least a few of the assays presented.*

We used known stressors in our studies, such as tellurite (metal inducing peroxidation), TBH (in replacement of H_2O_2 because *B. cenocepacia* has highly active catalase/peroxidase enzymes and TBH is a peroxide form that is resistant to catalase activity, as explained in the text; **see RV, lines 249-250**), cold (physical condition that is well known in plants to be associated with increased lipid peroxidation; **see RV, lines 224-225**).

4. Lipidomics: In their lipidomics experiments, the authors find no significant changes in the amount of PE, but increased levels of CL and PG, as a result of polymyxin B treatment (Fig. 9). This appears partially similar to a previous observation in ref. 24, where the authors claimed larger CL/PE ratios after treatment with ciprofloxacin, kanamycin, and ampicillin in E. coli. However, in that work, PG does not seem to be significantly increased as a result of treatment. Could the authors better compare and contrast their lipidomics results to these and other results present in the literature?

In the study highlighted by the reviewer and referenced in the manuscript, the authors quantified the ratio of CL/PE in bacterial membranes after antibiotics insults using the semi-quantitative thin layer chromatography method. In our

study, we used HPLC-MS for lipidomics, which provides better sensitivity, but more importantly allowed us to quantify the changes of each individual lipid species using an internal standard (SPE). Therefore, we could test whether polymyxin treatment had an impact on CL or PE since the increase of CL/PE ratio as shown in ref 24 could be due to either increase in CL or a decrease in PE. Our results **(RV, S11 Fig)** indicate that polymyxin treatment affected CL but not PE. We also enclose a figure below for the reviewer in which we expressed our results in CL/PE ratios; the data indicate that polymyxin only affected the CL/PE ratios in the WT and possibly in the BcnA but not in the other two mutants. This is consistent with our idea that anionic phospholipids including cardiolipin may be primarily targeted by peroxidation damage over PE.

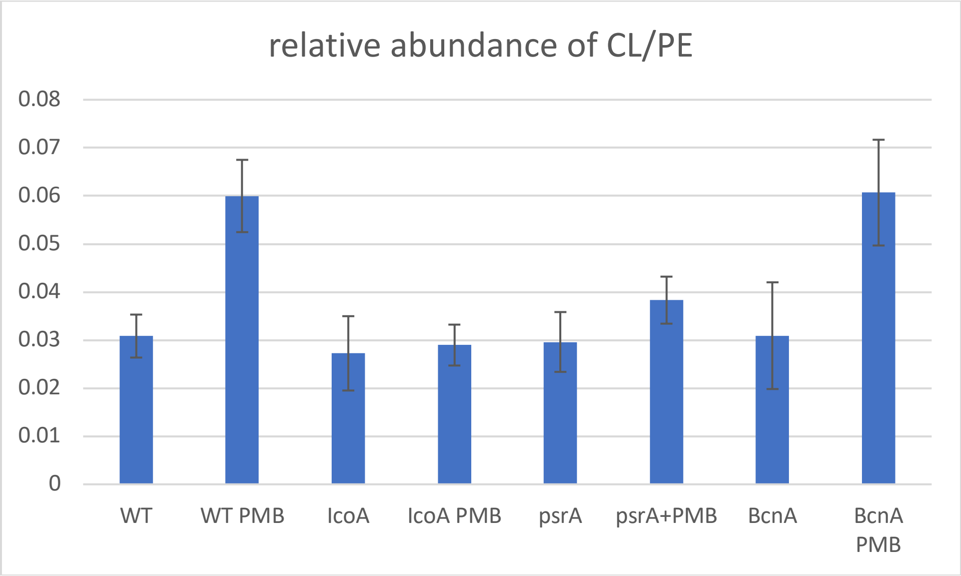

Additional points:

1. Line 47: This line needs to be rephrased, as not all toxic reactive byproducts are reactive oxygen species (see, for instance, reactive nitrogen and electrophilic species).
The reviewer is correct; we have added this information in the opening sentence of the **RV, lines 47-48**.

2. Paragraph beginning on line 56: the focus on Gram-negatives is appropriate, but begs the question of what happens in Gram-positives. It would be helpful to readers if the authors could add a sentence or two mentioning this.
Agreed; we have added modifications in the **RV; see lines 57-58, 76**.

3. Line 72: I do not think "near-lethal" is correct, since at least one of the references (ref. 24) apply antibiotics at both sub-MIC and substantially lethal concentrations.
The maximum effect on peroxidation is apparent at concentrations approaching lethality, but for consistency and clarity we have changed near lethal to sublethal throughout the RV.

4. Line 148: Specifying the species again here would help the reader.
Done; see **RV line 145**.

5. Line 194: Please name the gene before describing the knockout, or revise the presentation of this paragraph. The indicated knockout strain name was nowhere to be found in Fig. 2!
The reviewer was correct; it appeared confusing as originally written. The text has been edited to reflect the new gene name, which has been introduced much earlier. See **RV, lines 186-188**.

6. Line 232, the reference should be to Fig. 2B.
This is now Fig. 1B; see **RV, line 227.**

7. Line 290, since the authors only measure MDA, "byproducts" should not be plural.
Corrected; see **RV, line 286**.

8. Fig. 3, are the MDA levels normalized to bacterial protein concentrations? The methods mention a Bradford assay, but no normalization is mentioned in the figure legend.

It is mentioned now in the legend to Fig. 2; see **RV, lines 215-216**.

9. Fig. S5, presenting the figures normalized to mean untreated intensities as they vary along the cellular axis would make it easier for readers to compare the treatment effects.
This is a very good point. We have normalized the fluorescence distribution along the axis of bacterial cells by the average fluorescence of the untreated control. The method has been explained in Material and Methods (**RV, lines 737-742**) and information was also added to the figure legend (**RV, S8 Fig**).

10. Fig. 6, what is the rationale for changing the concentration to 75% MIC here? As mentioned above, the authors should perform dose-response experiments to confirm these results across a range of concentrations.
75% MIC was out standard treatment for WT and mutants to allow for growth under antibiotic stress using sublethal amounts of antibiotic according to the MIC of each mutant that was lower than that of WT. **Fig 6 is now S5 Fig in the RV.**

11. Figs. 5, 6, and 8 and associated SI figures need scale bars. Also, the units of pixel^2 seem incorrect.
Scale bars have been added to all figures in the RV. The units of $pixel^2$ were also presented with same scales in the same figures.

12. For visual clarity, Fig. 7 might be better presented as fluorescence and phase separately. The authors could add the overlay in addition to these two.
The fluorescence emitted by H_4BPMHC is slightly weaker and the cell bodies are not so obvious from the fluorescence channel. The overlay with phase allows for a better indication of the fluorescence distribution in the cell body of the bacterial cells. **This figure is now S9 Fig in the RV.**

13. Some of the data for K56-2 in Figs. 6, S3, S4, and S6 seem inconsistent with those presented in Fig. 5. Are these the same data, and if not, why is the former mean greater than the latter mean? Additionally, some of the K56-2 data in Fig. 8 seem awfully similar to the K56-2 data in the other figures, but Fig. 8 uses a different fluorescence probe. Could the authors please clarify?
Concerning the first part of the comment, the source of confusion stemmed from the use of different scales in the y axis of the graphs, which has been now corrected for all figures in the RV. As for Fig.8 **(now Fig4 in the RV)**, we do not understand what the reviewer implies. This figure corresponds to a different experiment from those using the green-fluorescent dyes and involves a very different dye that stains anionic phospholipids.

14. The CFU/mL ranges in Figs. 2 and 4 seem somewhat small, as they only span at most one log. These differences may be smaller than what some readers might be used to, for instance when examining treatment of other pathogens like E. coli and S. aureus with antibiotics (where differences in survival are typically at least one, if not multiple, logs). Perhaps the authors could comment on this difference if they feel it could improve the accessibility of their work.
B. cenocepacia is known to be susceptible to cold. In general, colonies on LB plates and stored in the fridge become not viable after a week. It is not surprising to us that the recovery is low, but still clear differences are present. We see similar results with *bcnA* mutants in *Klebsiella* and *Enterobacter cloacae* which we have recently constructed, but we these results are part of a different study beyond the scope of this one.

Comments from Academic Editor Bert van den Berg:

We thank Professor van den Berg's comments indicating our work is novel, interesting and opens new avenues of research. Concerning the length of the manuscript for a Discovery Report, our manuscript was intended as a full research article. Upon submission it was decided by the PlosBiology editorial team to proceed to review with the submitted version with the understanding that the manuscript would be consider as a Discovery Report.

Concerning the other questions and comments:

1. Since I'm not very familiar with the topic, I was wondering how important the prevention of lipid peroxidation is under "regular" conditions. It seemed to me that the chemicals and conditions used to induce the effects of BncA etc are somewhat exotic. Can the authors make a reasonable argument that the main role of these proteins is indeed to limit the harmful effects of lipid peroxidation?
This is an excellent point. BcnA was discovered by us as a protein of unknow function during a research project investigating bacteria heteroresistance to antibiotics. This is a situation poorly understood mechanistically but commonly associated to high levels of intrinsic resistance whereby a seemingly isogenic bacterial population possesses mix populations of bacteria with different levels of resistance including higher than the MIC of the

population (which is only an average value after all). We also found that polyamines are relevant in this context. The absence of reported function for bacterial lipocalins prompted us to investigate more and we realised that these proteins could be involved in providing a means for bacteria to withstand peroxidation. Due to the location of the protein in the periplasm (often secreted too in the case of Burkholderia), the presence of a ubiquinone in other bacterial BcnA homologues that were crystallized, and the association with a membrane cytochrome suggested to us that this set of proteins could form a system to provide a level of protection by detoxification of radicals formed in the outer membrane under extreme stress conditions such as bacterial exposure to sublethal and lethal antibiotic concentrations. We are currently investigating the function of the quinone by a combination of mutagenesis, chemical probes and molecular modelling and starting to characterize the cytochrome in detail. We propose that the quinone in the lipocalin is oxidised by the hydroperoxyl radicals and the cytochrome can reduce it, so the cycle starts again, and the two proteins work together as a peroxyl radicals sink. Any other products, like MDA are membrane permeable and they can be dealt with by aldehyde reductases such as PsrA. We allude to this model the end of our discussion in this manuscript, but we would like to have more solid evidence before we can present it in a more forceful way to the scientific community.

2. *line 83: what is being scavenged by the lipocalins? Do the authors think it is feasible in future work to determine directly (eg by mass spec) what kind of compounds are bound under various conditions?*
We think that lipocalins bind ubiquinone and are currently performing experiments, including MS to validate this. Please refer to the **Discussion in the RV, lines 471-473 and 487-495**.

3. *line 109: "co-regulated". Is this up- or down-regulation or either, depending on conditions?*
The genes are upregulated under antibiotic pressure and paraquat (methylviologen), a pesticide that induces endogenous hydroxyl radicals' formation. We have changed the text to "upregulated"; **RV, lines 109-110.**

4. *line 123: do LcoA/BncA/B orthologs occur in different phyla than the Proteobacteria?*
BcnA/B occur in most bacteria including Gram-positives. The main difference is the Gram-positive orthologues lack a signal peptide, suggesting that the proteins function in the bacterial cytoplasm. We have some suggestions based on bioinformatics that in *S. aureus, the* lipocalin may work in concert with lipid synthesis pathways but have not done any studies towards this end. We mainly analysed Proteobacteria to be keep the focus on the story of the manuscript since a detailed phylogenetic analysis encompassing all phyla would be outside of this work

5. *Given that orthologs exist in "model" bacteria, why do the authors perform these experiments in an exotic bug like Burkholderia cenocepacia?*
As indicated in our response to comment 1, we discovered the system in *B. cenocepacia*. This microorganism is a model opportunistic pathogen. In enteric bacteria such as *E. coli*, there are 2 lipocalins: a BcnA homologue and a second one, called Bcl, which have been only partially studied and proposed to be a phospholipid transported but with very little direct evidence of this function. We are extending our research onto enteric bacteria (*Klebsiella* and *Enterobacter*) to directly establish the function of the two proteins.

6. *line 157: does the DPPP=O fluorescence originate from the IM, OM or both and does this matter?*
DPPP=O fluorescence originate from any membrane since the probe requires a hydrophobic environment. It would matter for us to eventually define that the peroxidation of the outer membrane lipids drives the system, since we expect that the outer membrane has less compensatory mechanisms to replace damaged lipids than the inner membrane, which is in contact to cytoplasmic enzymes, etc. At present, this is the best we can do with this probe. We would require a probe that differentially integrates in one membrane or the other, which to our knowledge is currently lacking.

7. *line 174: are the cells fine at higher temperatures? What is the relevance of doing the experiments at 8 degrees C given that orthologs are also present in Enterobacteria that are unlikely to be exposed to such low temperatures?*
We explained in the manuscript that peroxidation is known to increase in plants under low temperatures and this process requires lipocalins that are from the same family as BcnA. We have indicated this in the **RV (see line 224-225)**.

8. *line 197 and methods: regarding the homology modelling, the authors should use something more sophisticated for this, i.e. AlphaFold.*
We examined the predicted structure of PsrA by de novo modelling using I-TASSER. This has been clarified in the **RV, lines 193-195** and in the Methods, **lines 753-761.**

line 232: "Fig. 3B" is wrong.
Also picked up by Reviewer 1; corrected.

9. *One thing that would be useful is to check the expression of the various proteins, with and without plasmid complementation. A failure to complement could be the result of a lack of expression. Or a complementation could be the result of a 10-fold higher expression than in wild type, for example.*
This is a good idea, but it would be difficult since we do not have specific antibodies to each of the native proteins and routinely employ epitope tags for detection. RNA studies would address this in part, but again, RNA and protein expression may not be in sync.

10. *line 313: why was 0.25 MIC used and not 0.5 like in other experiments (line 280)?*
The reason is that *B. cenocepacia* is an obligate aerobe and no growth of mutants and WT could be seen at concentrations of norfloxacin higher than 0.25 MIC; **See RV, lines 290-292**.

11. *An important issue is the selection of cells for the various fluorescence data from 5-8. How do you avoid (conscious or unconscious) cherry picking of bacteria that show what you want? The methods were not clear on this. It is really crucial to avoid selection bias since the differences between the various strains are not that big. Also, subtle differences between various panels are sometimes hard to see in the version I downloaded.*
Most experiments were performed independently by 2 individuals (authors MN and NF) and other by 3 (MN, NF, and PZ). Also, the quantitation was performed with multiple images to reduce selection bias. **See RV, lines 712-713.**

12. line 388: I assume the "antioxidant scavenging" refers to that in solution?
Correct; **See RV line 354**.

13. line 425: why would the loss of BcnA, LcoA and PsrA result in the accumulation of anionic phospholipids? To replace peroxidised ones?
We suggest that the peroxidation targets anionic phospholipids and the cell responds by increasing their amount. As described this section was not unclear. We have made several edits in the **RV; see lines 417-438**.

14. *line 435: "higher affinity". Higher than what?*
This was a typo; we meant "high:; **See RV, lines 392**.

15. *Line 461: "quantitative lipidomics to determine the overall lipid content". Why don't you try and detect peroxidised lipids directly by MS? What kind of compounds do you expect? It would be great to measure peroxidation directly rather than indirectly with various fluorogenic probes.*
We expect multiple rounds of peroxidation will produce a range of reactive aldehydes (including malondialdehyde) leading to lipid degradation. If a lipid experiences multiple rounds of oxidation, it is degraded and will not be captured by lipidomics since this solvent extraction methods only captures native intact lipids, such as PG, PE, and CL as we have shown. However, we are exploring way to capture the peroxidised lipids by optimising the extraction protocols

16. *line 471: replace :"under no" by "without".*
done; **See RV, line 436**.

17. *The discussion seems overly long and could probably be condensed.*
We have edited the discussion eliminating repetition and reducing the text to about 2/3 of the original version.

We have also introduced minor edits to the original version to improve clarity, reduced repetition and rearranged some sections in view of the new distribution of figures. As a Discovery Report, the main text has only 4 figures and there 11 supplementary figures plus 3 supplementary tables. We do not have Western blots or gels in this work.

Overall, we thank both reviewers and the technical editorial team for their help improving our manuscript and hope it now meets the standard for acceptance to PlosBiology.

Miguel A. Valvano
